# APOE ε4 moderates abnormal CSF-abeta-42 levels, while neurocognitive impairment is associated with abnormal CSF tau levels in HIV+ individuals – a cross-sectional observational study

**DOI:** 10.1186/s12883-015-0298-0

**Published:** 2015-04-01

**Authors:** Lucette A Cysique, Timothy Hewitt, Juliana Croitoru-Lamoury, Kevin Taddei, Ralph N Martins, Constance SN Chew, Nicholas NWS Davies, Patricia Price, Bruce J Brew

**Affiliations:** University of New South Wales, St. Vincent’s Hospital Clinical School, Sydney, Australia; Neuroscience Research Australia, Sydney, Australia; Department of Neurology St. Vincent’s Hospital, Sydney, Australia; St. Vincent’s Centre for Applied Medical Research, Sydney, Australia; University of Notre Dame, Sydney, Australia; Centre of Excellence for Alzheimer’s disease Research & Care, School of Medical Sciences, Edith Cowan University, Mount Lawley, Australia; Sir James McCusker Alzheimer’s Disease Research Unit, Hollywood Private Hospital, Nedlands, Australia; School of Psychiatry and Clinical Neurosciences, University of Western Australia, Perth, Australia; School of Biomedical Science, Curtin University of Technology, Bentley, Australia; Department of Neurology, Chelsea & Westminster Hospital, London, UK

**Keywords:** HIV/AIDS, CSF, APOE, Aβ1-42, t-tau, p-tau, HIV-associated neurocognitive disorders

## Abstract

**Background:**

Cerebrospinal fluid (CSF) biomarkers Aβ1-42, t-tau and p-tau have a characteristic pattern in Alzheimer’s Disease (AD). Their roles in HIV-associated neurocognitive disorder (HAND) remains unclear.

**Methods:**

Adults with chronic treated HIV disease were recruited (n = 43, aged 56.7 ± 7.9; 32% aged 60+; median HIV duration 20 years, >95% plasma and CSF HIV RNA <50 cp/mL, on cART for a median 24 months). All underwent standard neuropsychological testing (61% had HAND), APOE genotyping (30.9% carried APOE ε4 and 7.1% were ε4 homozygotes) and a lumbar puncture. Concentrations of Aβ1-42, t-tau and p-tau were assessed in the CSF using commercial ELISAs. Current neurocognitive status was defined using the continuous Global Deficit Score, which grades impairment in clinically relevant categories. History of HAND was recorded. Univariate correlations informed multivariate models, which were corrected for nadir CD4-T cell counts and HIV duration.

**Results:**

Carriage of APOE ε4 predicted markedly lower levels of CSF Aβ1-42 in univariate (r = -.50; *p* = .001) and multivariate analyses (R^2^ = .25; *p* < .0003). Greater levels of neurocognitive impairment were associated with higher CSF levels of p-tau in univariate analyses (r = .32; *p* = .03) and multivariate analyses (R^2^ = .10; p = .03). AD risk prediction cut-offs incorporating all three CSF biomarkers suggested that 12.5% of participants had a high risk for AD. Having a CSF-AD like profile was more frequent in those with current (*p* = .05) and past HIV-associated dementia (*p* = .03).

**Conclusions:**

Similarly to larger studies, APOE ε4 genotype was not directly associated with HAND, but moderated CSF levels of Aβ1-42 in a minority of participants. In the majority of participants, increased CSF p-tau levels were associated with current neurocognitive impairment. Combined CSF biomarker risk for AD in the current HIV+ sample is more than 10 times greater than in the Australian population of the same age. Larger prospective studies are warranted.

**Electronic supplementary material:**

The online version of this article (doi:10.1186/s12883-015-0298-0) contains supplementary material, which is available to authorized users.

## Background

The cerebrospinal fluid (CSF) biomarkers Aβ1-42, t-tau and p-tau have a characteristic pattern in Alzheimer’s Disease (AD) (AD profile: low levels of Aβ1-42 and high levels of t-and p-tau [1,2]) and may be associated with the cognitive changes seen in people with HIV-associated neurocognitive disorder (HAND). Links between AD and HAND are becoming increasingly relevant as AD is generally associated with age, and HIV+ individuals now live longer on effective combined antiretroviral therapy (cART). Also, there is some preliminary evidence for accelerated aging of the brain in middle-aged HIV+ individuals [3]. Three studies have investigated a potential link between these two disease entities. Brew and colleagues found an AD-like pattern of CSF Aβ1-42, t-tau and p-tau concentrations in mild to moderate HAND (N = 87; 46 AIDS-dementia complex (ADC) stage 1; 41 ADC stage 2; age: μ = 42; SD = 11) consistent with that seen in AD [4]. A study in HIV+ individuals with HAND (N = 21; 3 ADC stage 1; 18 ADC stages 2-4; age: μ = 38; SD = 18;) reported low levels of Aβ1-42 but normal levels of t-tau and p-tau [5]. Another study (N = 49; 30 MCD; 11 HAD; 8 unknown; age: μ = 48; SD = 8) reported low levels of Aβ1-42 and t-tau and p-tau [6]. These inconsistent results may reflect heterogeneity in the severity of HAND, the efficacy of cART, HIV disease duration and/or patient age.

Importantly, no study included APOE (Apolipoprotein E) genotyping, which may affect AD independently, or in association with, CSF biomarkers depending on the age of the subject [7]. The ε4 allele of APOE, an apolipoprotein thought to be partially responsible for amyloid clearance in the CNS, is one of the greatest known risk factors for late-onset sporadic AD [8,9]. Large cohort studies have provided conflicting results regarding the role of APOE in HIV+ persons with HAND. A study based on the Hawaii Aging with HIV Cohort [10] (N = 182) associated APOE ε4 carriage with HAND, but only in older participants. The largest study conducted in an ethnically diverse cohort [11] (Wilford Hall Medical Center, WHMC; 1,267 HIV-seropositive adults and 1,132 ethnically similar HIV-seronegative controls) found an association between APOE ε4/ε4 genotype and acceleration of HIV disease, but not with HAD. However the authors only assessed HAD rather than the complete HAND spectrum. Moreover, it is not clear how HAD was defined and assessed. More recently the CNS HIV Antiretroviral Therapy Effects Research (CHARTER) study [12] of 466 HIV+ participants (mean age = 44) who received a comprehensive HAND assessment showed no association between APOE ε4 carriage and HAND. They similarly found no association when they restricted their analyses to those with moderate HAND (Mild Neurocognitive Disorder (MND) and HAD). Age did not influence their results, but their sample included only 3.7% of persons aged 60+, compared to 25% in the Hawaii Aging with HIV Cohort [10]. The authors conclude that within the age-group they investigated APOE was not associated with HAND, confirming other smaller studies in same-age or younger samples [13-15], but stated that their results does not “preclude emergence of an association between APOE status and HAND as this population ages”, so further studies are needed.

The current study included 43 chronic HIV+ adults aged 56.7 ± 7.9 (32% aged 60+) years on long-term cART with no detectable HIV RNA in their plasma or CSF. The aims were: 1. Investigate whether the biomarkers Aβ1-42, t-tau and p-tau follow a similar pattern to that found in AD and assess the prevalence of CSF-AD like profile. 2. Investigate the relationship between APOE genotype, CSF biomarker levels and severity of HAND in these individuals. We hypothesized that HIV+ adults with HAND would be more likely to have a CSF AD-like profile than those who did not have HAND. Our second aim was exploratory, as APOE genotypes have not ben correlated CSF markers in chronic HIV infection.

## Methods

### Study participants

Participants were recruited through the HIV and Neurology Clinics at St Vincent’s Hospital, Sydney, Australia. Eligibility criteria included: age ≥45 years, stable cART ≥6 months, nadir CD4 T-cells ≤350/ul and HIV duration ≥5 years. Exclusion criteria included a history of non-HIV related neurological disorders or uncontrolled axis I psychiatric disorders, history of psychotic disorder, substance/alcohol use disorders (DSM-IV) within 12 months of enrolment, history of loss of consciousness >30 minutes, and non-proficiency in English (published protocol details [3,16]). St. Vincent’s Hospital, The University of New South Wales, and the University of Western Australia Human Research Ethics Committees approved protocols. All participants provided written informed consent.

### Current impairment status

Detailed testing procedures have been published Lane et al. [16] and Cysique et al. [17]. Briefly, HIV+ participants underwent a standard neuropsychological evaluation assessing seven cognitive domains. Impairment status was determined using local normative standards (z-scores) developed in a demographically comparable HIV- sample recruited as part of the HIV and Brain Aging research program at the University of New South Wales (PI, LAC). Details on methods and the sample characteristics used to develop local norms have been published [17]. The standard Global Deficit Score (GDS) method [18,19] was used to classify impairment. As per convention [18,19], GDS ≥ 0.5 was used to define a clinically relevant level of impairment yielding a discrete outcome (impaired or unimpaired) or a continuous outcome. A higher GDS indicates greater impairment. The standard GDS cut-off of ≥0.5 is widely used to assess HIV-related brain injury and meets the Frascatti criteria for HAND [20]. For each participant, we determined the HAND classification (Asymptomatic Neurocognitive Impairment: ANI; Mild Neurocognitive disorder: MND; or HAD) as follows: [20]. GDS ≥ 0.5 & no Independence in Activities of Daily Living (IADL) [21] decline = ANI; GDS ≥ 0.5 & mild/moderate IADL decline = MND; GDS ≥ 1.5 & severe IADL decline = HAD. IADL information was obtained from a standard IADL scale, the Patients Assessment of Own Functioning Inventory [22] and any clinical evidence of IADL decline (medical record; nurse information).

### Past HAND status

History of HAND was based on standard neurological and neuropsychological examinations, MRI/MRS scans and an extended panel of plasma and CSF biomarkers, as described previously [23]. LC consulted the medical records and recorded HAND diagnoses reported using the AIDS Dementia Complex nomenclature [24]. This was adapted to the HAND 2007 criteria [25] by LC (see Additional file 1) and reviewed by BJB (HIV Neurologist) to reach clinically relevant diagnoses of MND and HAD [25]. Past HAND status was also analysed as a dichomotous variable.

### Specimen collection and quantitation of biomarkers in cerebrospinal fluid

**Figure 1:**
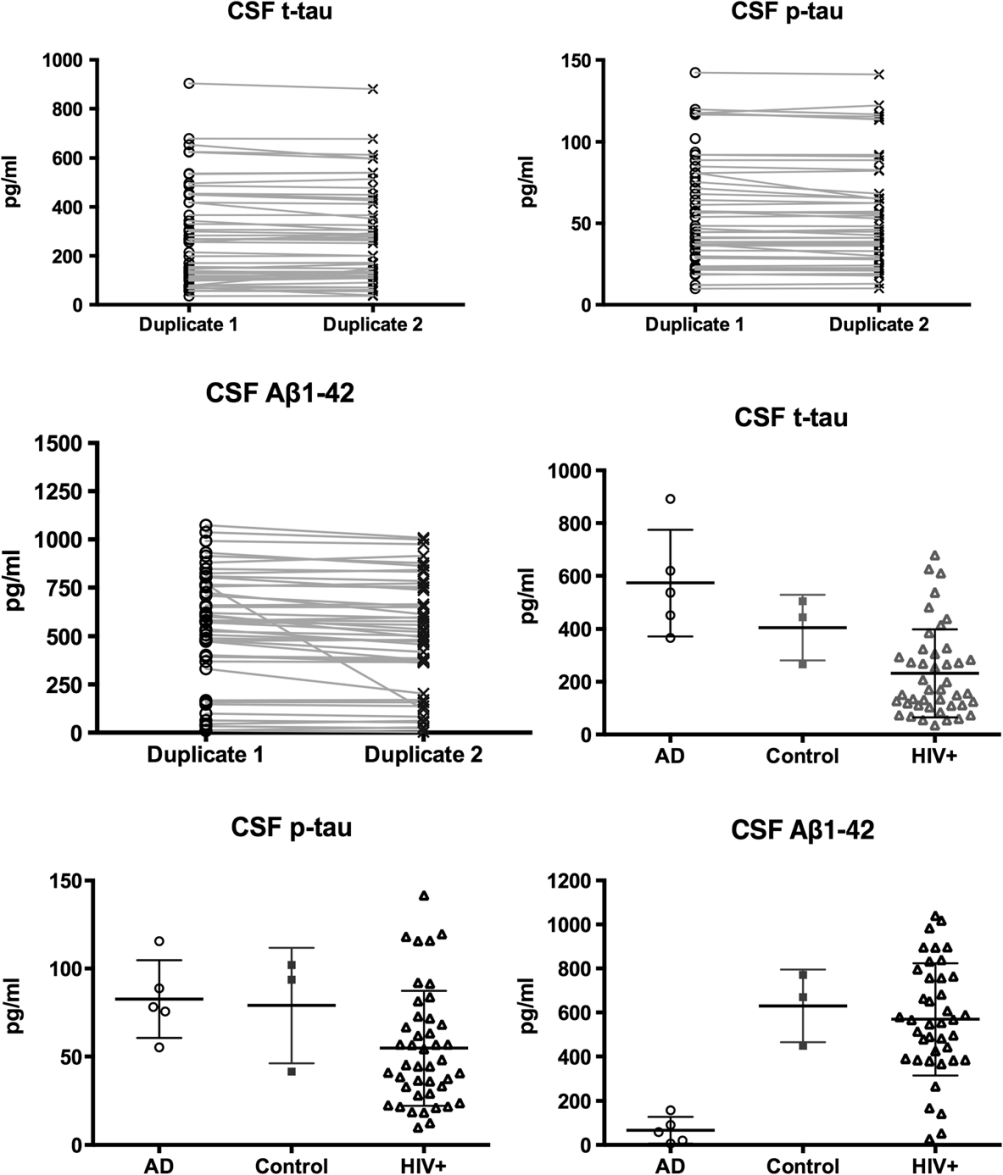
**Raw duplicate and average data for the CSF biomarkers**. To assess the reliability of all the CSF biomarker concentrations, we performed correlations between assays duplicate for t-tau, p-tau (r = .99; p < .0001) and Aβ1-42 (r = .95; p < .0001). They were highly reliable. To assess the within group duplicate reliability we conducted a *t*-test between duplicate 1 and 2 within HIV, AD and HIV- group and all results were non-significant differences (t-tau; p-tau: *p* > .90; Aβ1-42 *p* > .50).

**Figure 2:**
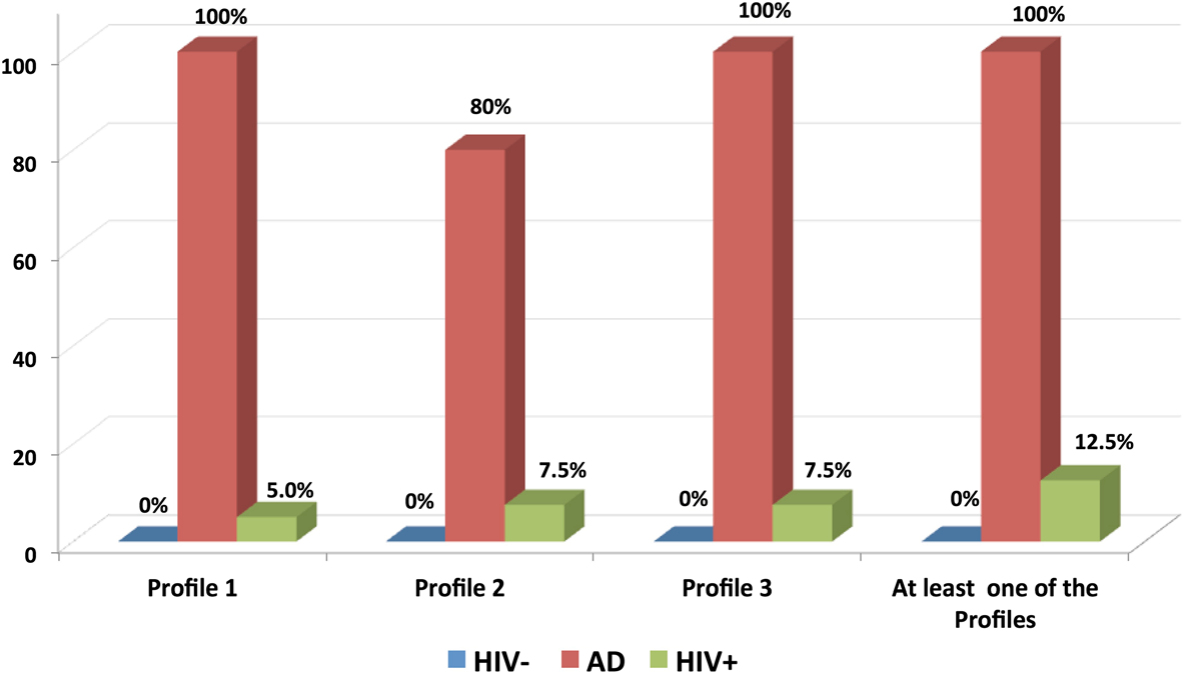
**Prevalence of clinically relevant CSF profiles**. CSF Aβ1-42 was missing for three cases because of insufficient CSF sample; therefore the CSF-AD like profiles were computed from 40 cases.

CSF was stored at -70°C until assayed. CSF Aβ1-42 concentrations were determined using a sandwich enzyme-linked immunoassay (ELISA) with a limit of detection of 15 pg/mL (Innotest™ β-amyloid_(1-42)_ ELISA, INNOGENETICS N.V, Ghent, Belgium). CSF t-tau and p-tau concentrations were determined using a sandwich ELISA with limits of detection of 87 and 15 pg/mL (Innotest™ hTAU Antigen assay and Innotest™ PHOSPHO-TAU_(181P)_ ELISA, INNOGENETICS N.V). As within-laboratory variation has been reported [26], we tested our procedure in a eight HIV- controls, comprising three cognitively healthy adults and five adults with AD recruited from the Memory Clinics of the Sir James McCusker Alzheimer’s Disease Research Unit (Western Australia) and the Neurology AD clinics at St. Vincent’s Hospital. AD diagnoses were based on the NINCS-ADRA [27] and DSM-IV [28] criteria. CSF samples were examined blind to the AD & HIV status and *vice versa*. CSF markers concentrations are presented in Figure 1. Laboratory analyses were reliable across duplicates (*p* < .0001). To examine the sensitivity and specificity of our assay procedure, we used published cut-offs [29] which display sensitivity (95%) and specificity (≥81%) for AD and incipient AD [30]: CSF-AD profiles were 1). t-tau >350 & Aβ1-42 < 530 pg/ml; 2). p-tau >60 & Aβ1-42 < 530 pg/ml; 3). t-tau >350 pg/ml & Aβ1-42/p-tau <6.5. At least one CSF-AD profile was found in all AD patients and none in the HIV- controls (see Figure 2). CSF Aβ1-42 was missing in three cases because there was insufficient CSF, so cut-offss were based on 40 cases.

### APOE genotyping

DNA was extracted from saliva or blood leukocytes using QIAamp DNA mini Kits (QIAGEN; Valencia, CA) and stored at -80°C. Two SNPs in the *APOE* gene (rs429358, rs7412) defining the *APOE* ɛ3, ɛ4 and ɛ2 variants were genotyped using TaqMan assays according to manufacturer’s instructions. Median (range) genotyping success rates were 99% (98%–100%).

### Data analyses

Categorical data were assessed with the Fisher’s Exact Test. Concentrations of t-tau, p-tau were log_10_ transformed to approximate normal distributions. Univariate analyses (Pearson and/or point biserial correlations) evaluated associations between the CSF biomarkers and AD risk factors [31] (APOE genotypes: homozygotes (ε4/ε4) vs. heterozygotes (ε4/ε2 or ε4/ε3) vs no ε4, and age), HIV-related parameters [32] (nadir CD4 T-cell count and HIV duration in years) and neurocognition (current GDS and past HAND status).

Multivariate analyses comprised stepwise regression models with CSF p-tau (t-tau was excluded as it correlated with p-tau) and Aβ1-42 separately as outcomes. For the Aβ1-42, predictors were included in the order defined above. For p-tau, the continuous GDS was entered first (based on univariate analyses), followed by past HAND status, age, HIV disease markers and APOE status. The model was re-run removing a priori APOE and past HAND status. We used a forward model selection with the Akaike Information Criterion corrected (AICc), as it is less reliant on p-value than other type of stepwise selection methods. The model with the lowest AICc yields the best compromise between goodness to fit and model complexity [33]. Statistical analyses were conducted using JMP 10 (SAS Inc).

## Results

Demographic and clinical characteristics of the HIV+ group are presented in Table 1, whilst characteristics of the reference samples are presented in Additional file 2. HIV+ participants were characterized by historic immune compromise, which had improved substantially on cART. Only 2 cases had detectable HIV RNA in plasma and one other in the CSF. These are considered to be “detection blips” as participants were clinically stable on cART and negative HIV RNA assessments had been recorded within the previous 12 months (data not shown). Most participants were well-educated white men. The AD and control sample were older than the HIV+ sample by design, but comparable for other demographics. They were included to demonstrate reliability of CSF assays and CSF-AD like profiling.

**Table 1:** **Clinical and laboratory characteristics in the entire HIV+ group**

	**HIV+ group only**	
Median Nadir CD4-T+ cells/μL	198	IQR: 31-279
Median current CD4-T+ cells/μL	597	IQR: 342-748
% Plasma undetectable HIV RNA (<50 cp/mL)^1^	95.5%	-
% CSF Undetectable HIV RNA (<50 cp/mL)^2^	97.6%	-
Median HIV duration (years)	20.5	IQR: 14-25
% AIDS status (CDC 1993)	70.5%	-
% With past AIDS defining illness	53%	-
Median current cART duration (months)	30	IQR: 12-54
% MSM as HIV risk factor	89%	-
Average educational level (years)	13.82	SD: 2.95
% Current hAND	61.3%	-
% Past hAND	31.8%	-
% APOE any ε4 allele carriers	30.9% (N = 13)	-
% APOE ε4/ε4 allele carriers	7.1% (N = 3)	-
% APOE ε2/ε3 allele carriers	7.1% (N = 3)	-
% APOE ε3/ε3 allele carriers	61.9% (N = 26)	-

IQR: Inter-quartile range.

SD: standard deviation.

MSM: Men who have sex with men.

HAND: HIV-associated neurocognitive disorders (Frascatti 2007 diagnostic criteria).

1. Two cases had detectable plasma viral load, which were considered blips, as these cases were undetectable before and after the HIV RNA testing concomitant to the current study neuropsychological testing. Not that those cases were undetectable in the CSF.

2. One case had detectable CSF HIV RNA and this was also considered a blip for the same reasons as for plasma HIV RNA. This case was undetectable in the plasma.

When the cut-offs distinguishing AD in HIV- and AD cases (described in methods) were applied, 5% (2/40) HIV+ participants had profile 1, 7.5% (3/40) had profile 2 and 7.5% (3/40) had profile 3. One participant met the AD-CSF profile on all 3 cut-offs. In total, 12.5% (5/40) had at least one CSF-AD like profile (Figure 2).

A diagnosis of HAND (versus GDS ≥0.5) did not associate with having a CSF AD- like profile (12.5% with or without HAND). However, those with HAD (40%) were more likely to have a CSF AD-like profile than those with ANI (0%; *p* = .05). They were also marginally more likely to have a CSD AD-like profile compared to with MND (16.7%), but this was not statistically significant. Participants with past HAND status were more likely to have at least one CSF AD-like profile than those with no past HAND (31% versus 3.7%; *p* = .03). Among those with past HAND, CSF AD-like profiles were marginally more common in HAD (37.5%) than in MND (20%) but this was not significant as there were only 8 HAD cases, 5 MND cases and 5 individuals with a CSF AD-like profile.

In univariate analyses (Table 2) APOE genotypes incorporating ε4 associated with lower levels of CSF Aβ1-42 (p=.001). Moreover the current level of neurocognitive impairment was associated with higher log_10_ levels of p-tau (*p* = .03) and t-tau (*p* = .05).

**Table 2:** **Correlations between individual CSF biomarkers, AD risk markers, HIV/HAND risk markers, current overall neurocognitive impairment and past HAND**

***Correlations between***		***r***	***p***
APOE	Log_10_ CSF t-tau	−0.09	0.59
Age		0.16	0.31
Nadir CD4		−0.19	0.22
HIV duration		0.04	0.78
Past HAND		0.10	0.50
GDS (current)		0.29	0.05
APOE	Log_10_ CSF p-tau	−0.11	0.47
Age		0.08	0.61
Nadir CD4		−0.18	0.26
HIV duration		−0.05	0.73
Past HAND		0.15	0.33
GDS (current)		0.32	0.03
APOE	CSF Aβ1-42	−0.50	0.001
Age		0.16	0.31
Nadir CD4		−0.09	0.58
HIV duration		−0.11	0.51
Past HAND		−0.06	0.70
GDS (current)		0.18	0.26
Log_10_ CSF p-tau	Log_10_ CSF t-tau	0.96	<.0001
CSF Aβ1-42	Log_10_ CSF t-tau	0.48	0.002
CSF Aβ1-42	Log_10_CSF p-tau	0.53	0.0004

APOE Genotypes were coded as follows: no ε4 = 1; heterozygotes ε4/ε2 or ε4/ε3 = 2; genotypes: homozygotes ε4/ε4 = 3. Note that the correlation for APOE is driven by the ε4/ε4 cases and one ε4/ε3 case.

We used Pearson and point-biserial correlations as appropriate.

The Global Deficit Score (GDS) is a summary score that is an average of all the deficit scores across the test battery, and it grades normal vs. impaired performance between 0-5. A higher GDS indicated greater *current* overall impairment.

Past HAND: History of HAND yes was coded 1; no was coded 0.

**Figure 3:**
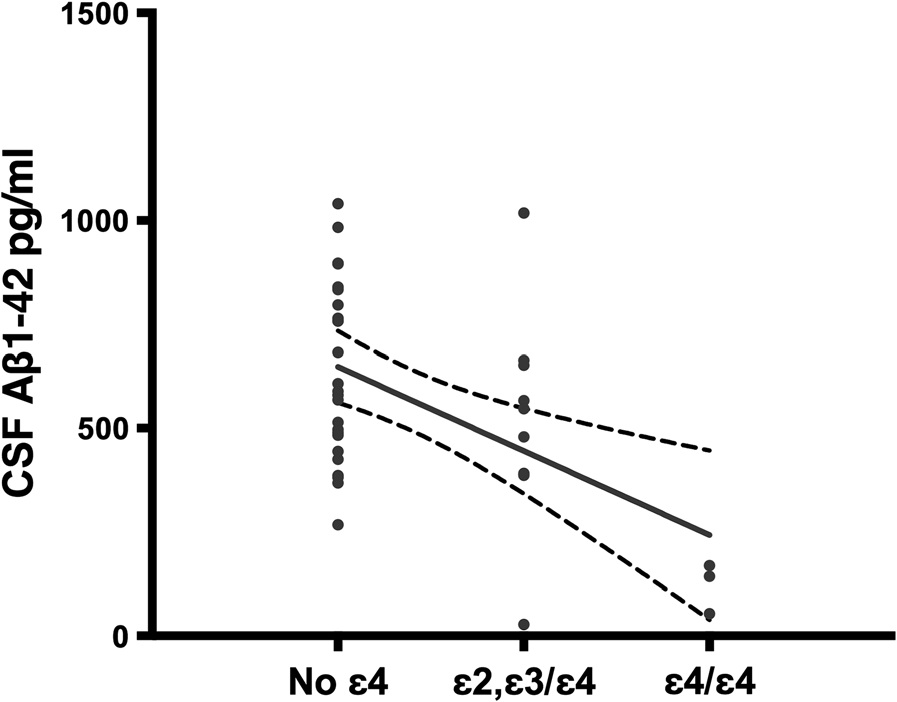
**Correlation between APOE status and CSF Aβ1-42.** Note that APOE genotyping is presented in Table 1.

In the multivariate model with CSF Aβ1-42 as the outcome, APOE ε4 (R^2^ = .25; *p* = .0003) and age (R^2^ = .07; *p* = .06) were optimal predictors based on the AICc criterion (model R^2^ = .32; AICc = 536). The APOE effect was driven by ε4/ε4 cases and one ε3/ε4 (Figure 3 represents the univariate correlation for clarity). The unusual positive and weak effect of age on CSF Aβ1-42 suggests a survivor bias in our cohort. This effect represents a non-significant small correlation in univariate analyses (see Table 2), but impacted the multivariate model.

**Figure 4:**
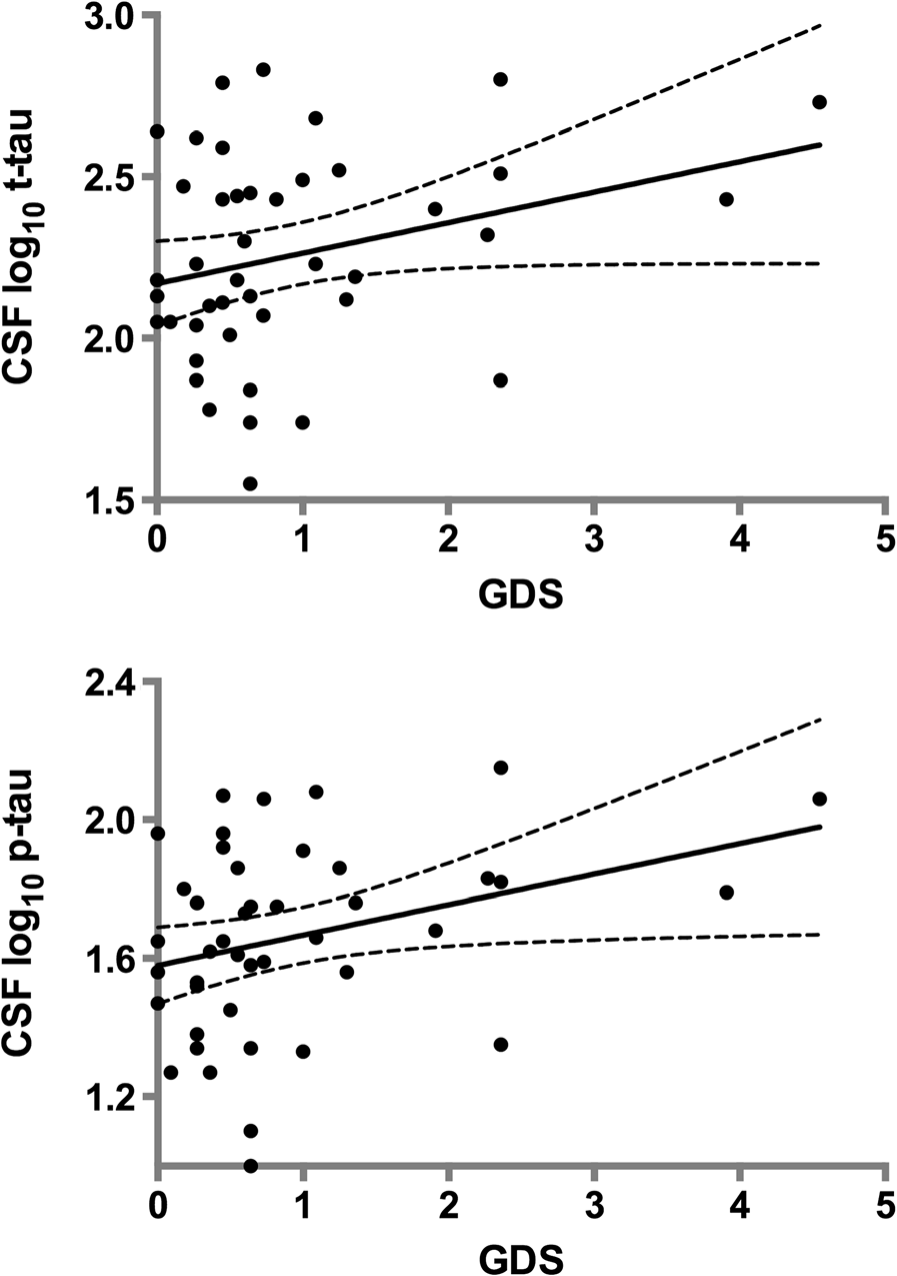
**Correlation between CSF p-tau, t-tau and neurocognitive impairment (continuous GDS).** A higher GDS indicates greater neurocognitive impairment. t-tau and p-tau were log_10_ transformed.

In the first multivariate model with CSF log_10_ p-tau as the outcome, and using all relevant study predictors, the AICc selected the GDS (*p* = .13) as the optimal predictor and the overall model was weak (model R^2^ = .05; AICc = 12) as correlations between predictors affected the direct explanatory power of the GDS. For example; the GDS was associated with past HAND (*p* = .0008) so the increase in p-tau reflects both current and past HAND (see Additional file 3). In the second run of the model, APOE and past HAND were excluded a priori, the GDS (*p* = .03) was selected by the AICc. However the overall model was only slightly improved, so the combination of factors still have a relatively weak explanatory power (model R^2^ = .10; AICc = 11.5). To highlight the effect of the GDS, we present the univariate correlation in Figure 4.

## Discussion

The study is the first to associate CSF AD-like profiles with current HAD, past MND and past HAD. While the number of cases with a CSF AD-like profile is small, this may be explained by poor survival over the years before cART was optimal. Despite this, it appears that HAD is either a risk for low CSF AD-like profile or the reverse – which would predict increases in HAD as the HIV+ population ages. Because our study is cross-sectional, it is unclear how HIV-related brain injury may have affected levels of CSF biomarkers, though this would be consistent with the impact of past HAND.

This study is the first to include both measurements of CSF neurodegenerative markers and APOE profiling in stable optimally treated HIV+ individuals. A novel finding was that APOE ε4 moderated CSF Aβ1-42 levels. In subjects without HIV, APOE ε4 carriage is associated with lower CSF Aβ1-42 concentrations [34]. This correlation emerges in subjects aged 46-65 years (the average age of our cohort) and is clearer in those aged 66-89 years. Similarly, HIV- homozygous carriers of the APOEε4 allele have lower CSF Aβ1-42 levels than heterozygote carriers [34]. Although our sample had a low APOE ε4/ε4 prevalence [cf: median 11.6%; range (4.9%–18.3)% in the Australian population:] [35], the effect was robust and maintained in multivariate analyses. As APOE genotype is a significant risk factor for AD [7] and affects post-mortem beta-amyloid burden in HAND [36], longitudinal studies are warranted.

Our other main finding was that neurocognitive impairment was associated with higher CSF p-tau and t-tau levels*.* This is not seen in normal aging. For example cognitively healthy individuals do not have elevated levels of p-tau and t-tau, but ~19% show abnormal CSF Aβ1-42 concentrations [34,37]. This could mean that p-tau and t-tau mark a greater risk for current HAND in middle-aged HIV+ individuals [38].

Lastly, past immune compromise and longer HIV duration may increase AD risk, as both are risk factors for HAND [39]. This was not evident here as the n value was low.

Our findings yield useful information for longitudinal study addressing neurodegeneration in stably treated HIV infection, and to in persons aged over 50-60 years. APOE genotyping and CSF biomarkers should be investigated as individual continuous outcomes as well as AD-like cut offs. This strategy will help determine the clinical relevance of a CSF neurodegenerative marker panel.

The inclusion of older participants at this stage of the HIV epidemic has some caveats as most are survivors of the pre-cART era. Thus our data may contain some survivor bias. Therefore until cohorts with no survivor bias (i.e.,: individuals started on cART) are available, the neuroHIV community should remain cautious of a lack of detection or weak signal of accelerated neurodegeneration in aging HIV+ persons. Such cohorts will display a lower prevalence of HAD but MND is still relatively common in persons on cART [17,20] and ANI can predict neurocognitive deterioration [40].

In addition CSF biomarkers and APOE genotype, other mechanisms might explain β-amyloid dysregulation in chronic HIV infection. Indeed our data suggests that higher CSF Aβ1-42 concentrations may correlate with CSF p-tau and t-tau levels. This is counter-intuitive as lower CSF Aβ1-42 and increased tau mark AD. However the inverse relationship was only seen in a sub-set of participants. In others CSF Aβ1-42 concentrations are normal or slightly elevated (Figure 1). Elevation of CSF Aβ1-42 concentrations may represent an anti-inflammatory effect [41]. Supporting this interpretation is the fact that CSF neopterin correlated with CSF Aβ1-42 (r = .40; p = .009).

## Conclusions

At first glance, our results seem contrary to studies which found no evidence of amyloid burden in HIV+ individuals [42] or no relationship between HAND and APOE ε4 genotype [12]. However we show that APOE ε4 genotype moderates the expression of CSF Aβ1-42 in participants with HAND. Moreover as in larger studies [29], we find that APOE does not directly impact HAND. Perhaps as most individuals are still relatively young (<60 years), amyloid build up is incomplete and tau pathologies may be a more common trigger for brain damage. With evidence that the CSF biomarker risk for AD in the current HIV+ sample is over 10 times greater (12.5%) than in the Australian population of the same age [43], this shows the need for a longitudinal study of the aging HIV+ population.

### Limitations

Our study offers new insights into the CSF biomarker pathologies in HIV infection, but the design is cross-sectional so the clinical consequences are unclear. Our study included a medium size sample, but the participants were well characterized and uniform in having achieved viral suppression on cART. This excludes HIV replication as a cause of HAND compared to previous studies [4-6]. Some APOE ε4 homozygotes may have died before cART was available (i.e., survival bias), as the prevalence of APOE ε4/ε4 is lower than in the Australian general population.

## Additional files

Additional file 1:
**Correspondence between the 1988 ADC and the 2007 American Academy of Neurology HAND diagnosAc nomenclature.**
Additional file 2: Table S1. Study groups’ demographic characteristics. Additional file 3: Table S2. Correlations between multivariate analyses predictors.

## Abbreviations

AD: Alzheimer’s disease
APOE: Apolipoprotein E
cART: combined antiretroviral therapy
CSF: Cerebrospinal fluid
ELISA: Enzyme-linked immunoassay
GDS: Global deficit score
HAD: HIV-associated dementia
HAND: HIV-associated neurocognitive disorder
HIV: Human immunodeficiency virus
MND: Mild neurocognitive disorder
t-tau: total tau
p-tau: phosphorylated tau
