# Supplementary material for: APOE ε4 moderates abnormal CSF-abeta-42 levels, while neurocognitive impairment is associated with abnormal CSF tau levels in HIV+ individuals – a cross-sectional observational study
Source: BMC Neurol. 2015 Apr 1;15:51. doi: 10.1186/s12883-015-0298-0 (PMC4386081; doi:10.1186/s12883-015-0298-0)
Supplement: Additional file 1: — Correspondence between the 1988 ADC and the 2007 American Academy of Neurology HAND diagnosAc nomenclature. [file 12883_2015_298_MOESM1_ESM.pdf]

# Correspondence between the 1988 ADC and the 2007 American Academy of Neurology HAND diagnostic nomenclatures

AIDS Dementia Complex (Price & Brew, 1988)		Today
Stage 0 (normal)	Normal mental and motor function	Normal
Stage 0.5 (subclinical)	Minimal or equivocal symptoms without impairment of work or activities of daily living (ADL). "Background" neurological signs, such as slowed fine finger movements or primitive reflexes, may be present.	ANI
Stage 1 (mild)	Cognitive deficit that compromises the performance of the more demanding aspects of work or ADL.	MND
Stage 2 (moderate)	Cognitive deficit makes the patient unable to perform work or the more demanding aspects of ADL. The patient may require assistance with walking.	HAD
Stage 3 (severe)	Cognitive deficit makes it possible for the patient to perform only the most rudimentary tasks, for example, the patient cannot follow news or sustain a conversation of any complexity. The patient often requires some support for walking.	HAD
Stage 4 (end-stage)	Cognitive deficit has reached the point where the patient has virtually no understanding of his or her surroundings and is virtually mute. The patient is paraparetic or paraplegic, often with double incontinence.	HAD

cART

Pre-cART

The rationale for the overlap and differences between the two nomenclatures is based on the evidence that cART has decreased the clinical severity of HAND (for review: **http://www.dovepress.com/management-issues-in-hiv-associated-neurocognitive-disorders-peer-reviewed-article-NBHIV**

cART: combined antiretroviral treatment; ANI: Asymptomatic Neurocognitive Impairment; MND: Mild Neurocognitive Disorder; HAD: HIV-associated Dementia.
