# Supplementary material for: APOE ε4 moderates abnormal CSF-abeta-42 levels, while neurocognitive impairment is associated with abnormal CSF tau levels in HIV+ individuals – a cross-sectional observational study
Source: BMC Neurol. 2015 Apr 1;15:51. doi: 10.1186/s12883-015-0298-0 (PMC4386081; doi:10.1186/s12883-015-0298-0)
Supplement: Additional file 2: Table S1. — Study groups’ demographic characteristics. [file 12883_2015_298_MOESM2_ESM.docx]

**Table S1: Study groups’ demographic characteristics**

|  | **HIV- elderly controls** | **HIV- AD** | **HIV+** |
| --- | --- | --- | --- |
| N | 3 | 5 | 43 |
| Mean Age (SD) | 64.3 (2.3) | 63 (5.8) | 56.7 (7.9) |
| Age range | 63-67 | 55-69 | 45-80 |
| Age > 60 years old | 100% | 60% | 32% |
| Sex (%male) | 100% | 100% | 98% |
| Caucasian background | 100% | 100% | 98% |

Note that participants were screened for current alcohol and substance use disorder (within 12 months of study entry). However, because history of alcohol and substance use disorder is prevalent in HIV+ persons and may affect tau levels [1], we determined whether cases with a history alcohol or substance use disorder (6/43; 14%) had different t-tau and p-tau levels compared to those without this history. In both cases, we found no differences in tau levels (for t-tau, p=.49; and for p-tau; p=.52). The six cases included: one with past alcohol use disorder; one with past alcohol and cocaine use disorder; one with past opiate use disorder; one with past methamphetamine use disorder; one with past with methamphetamine and cocaine use disorder and one with past polysubstance use disorder.

1. Ramage SN, Anthony IC, Carnie FW, Busuttil A, Robertson R, Bell JE. Hyperphosphorylated tau and amyloid precursor protein deposition is increased in the brains of young drug abusers. Neuropathol Appl Neurobiol. 2005;31(4):439-48.
