# Supplementary material for: APOE ε4 moderates abnormal CSF-abeta-42 levels, while neurocognitive impairment is associated with abnormal CSF tau levels in HIV+ individuals – a cross-sectional observational study
Source: BMC Neurol. 2015 Apr 1;15:51. doi: 10.1186/s12883-015-0298-0 (PMC4386081; doi:10.1186/s12883-015-0298-0)
Supplement: Additional file 3: Table S2. — Correlations between multivariate analyses predictors. [file 12883_2015_298_MOESM3_ESM.docx]

**Table S2: Correlations between multivariate analyses predictors**

| **Correlations between** | | ***r*** | ***p*** |
| --- | --- | --- | --- |
| Age | APOE | 0.20 | 0.21 |
| Nadir CD4 | APOE | -0.00 | 0.98 |
| Nadir CD4 | Age | 0.02 | 0.91 |
| HIV duration | APOE | 0.22 | 0.15 |
| HIV duration | Age | 0.09 | 0.58 |
| HIV duration | Nadir CD4 | -0.25 | 0.11 |
| Past HAND | APOE | 0.00 | 0.98 |
| Past HAND | Age | -0.06 | 0.70 |
| Past HAND | Nadir CD4 | -0.09 | 0.57 |
| Past HAND | HIV duration | -0.00 | 0.98 |
| GDS (current) | APOE | -0.08 | 0.59 |
| GDS (current) | Age | 0.13 | 0.41 |
| GDS (current) | Nadir CD4 | 0.03 | 0.84 |
| GDS (current) | HIV duration | -0.20 | 0.20 |
| GDS (current) | Past HAND | 0.49 | 0.0008* |

Pearson and point-biserial correlations were used as appropriate.

APOE Genotypes were coded as follows: no ε4 =1; heterozygotes ε4/ε2 or ε4/ε3=2; genotypes: homozygotes ε4/ε4=3.

The Global Deficit Score (GDS) is a summary score that is an average of all the deficit scores across the test battery, and it grades normal vs. impaired performance between 0-5. A higher GDS indicated greater *current* overall impairment.

Past HAND: History of HAND yes was coded 1; no was coded 0.
